# Attrition among HIV positive children enrolled under integrated HIV care programme in Myanmar: 12 years cohort analysis

**DOI:** 10.1080/16549716.2018.1510593

**Published:** 2018-09-07

**Authors:** Aung Chan Minn, Nang Thu Thu Kyaw, Thet Ko Aung, Ohn Mar Mon, Thurain Htun, Myo Minn Oo, July Moe, Aye Aye Mon, Srinath Satyanarayana, Htun Nyunt Oo

**Affiliations:** a International Union Against Tuberculosis and Lung Disease, Myanmar country office, Mandalay, Myanmar; b Ministry of Health and Sports (MOHS), 550 bedded Children Hospital, Mandalay, Myanmar; c Center for Operational Research, International Union Against Tuberculosis and Lung Diseases, Paris, France; d National AIDS Program, Department of Public Health, Ministry of Health and Sports, Nay Pyi Taw, Myanmar

**Keywords:** Attrition, death, lost to follow up, retention, children, HIV, SORT-IT

## Abstract

**Background**: In Myanmar, HIV seropositive children are being enrolled in an integrated HIV care (IHC) Program for HIV treatment and care since 2005.

**Objectives**: To assess the: (a) attrition (death or loss-to-follow-up) rates among children (aged ≥ 18 months to < 15 years) enrolled into the programme before and after initiation of anti-retroviral therapy (ART) (pre-ART and ART periods); (b) demographic and clinical factors associated with attrition during these two periods.

**Methods**: Children enrolled in IHC Programme and their status (death, lost to follow-up, regular follow-up or transferred out) was assessed as on 30 June 2017. Attrition rates (per 100 person-years) at pre – ART and ART periods were calculated and the association between demographic and clinical characteristics with attrition was assessed using Cox proportional hazards model.

**Results**: Among 2,736 children enrolled, pre-ART attrition rate was 19 per 100 person-years of follow-up (95% CI: 17–21) and ART attrition rate was 4 per 100 person-years of follow-up (95% CI: 3–4) with higher levels during the initial few months of enrolment. The 36-month retention rates during pre-ART period was 75% (95% CI: 72–78) and during ART period was 87% (95% CI: 86–88). The children ‘at enrolment’ with relatively lower levels of haemoglobin, immune deficiency, underweight for age, higher WHO clinical stages, presence of hepatitis B infection had higher hazards of attrition in both periods.

**Conclusion**: The attrition rates are high particularly among children with relatively poorer clinical, nutritional profiles at enrolment. The study suggests the urgent need for improving adherence counselling especially during the initial few months of enrolment and early ART initiation.

## Background

Globally, 2.1 million children aged < 15 years were living with Human Immunodeficiency Virus (HIV) in 2016 and among them 120,000 experienced AIDS-related death [1]. Early detection of HIV infection, linking and retaining them on anti-retroviral therapy (ART) is essential for reducing the morbidity and improving survival. However, globally only 43% of children living with HIV received ART in 2016 [2] and the attrition (loss to follow-up and/or death) of children on ART is higher in resource limited settings when compared to developed countries [3,4]. Many studies in Africa and Asia have shown that it is challenging to enrol and retain paediatric patients on ART care [5–7]. A study from South Africa showed that only 71% of HIV-positive infants were linked to HIV care [8]. A systematic review on children living with HIV showed that on an average the 36-month retention on ART was 66% in Africa and 74% in Asia [9]. This systematic review highlighted that there was limited data from Asia region and pointed out that there was no data from Myanmar, the country with one of the largest HIV burden in South-East Asia.

Myanmar has a concentrated epidemic of HIV. The HIV prevalence rate in the general population in 2016 was 0.53% (~ 230,000 persons) [10]. Of these, it was estimated that 9,300 were children aged < 15 years of which 7,300 were receiving ART [10]. The Integrated HIV care (IHC) programme in Myanmar started in public sector in collaboration with Ministry of Health and Sports, National AIDS Programme and, National Tuberculosis Programme has been providing comprehensive HIV care for both adults and children since 2005. By the end of 2016, the IHC programme had enrolled more than 38,000 adults and 3,000 children for HIV care.

A couple of studies using adult HIV seropositive patient data (aged ≥ 15 years of age) from IHC programme have shown that the attrition rates in pre-ART care and ART care is 32% and 16%, respectively (5-year cohort analysis with 13.7 months median duration of follow-up) [5,11]. Compared to these results, private sector adult ART cohort in Myanmar had a relatively higher attrition rate of 20% during 5-years follow-up [12]. A study conducted on a cohort of adult patients receiving ART from general practitioners showed that ~ 12% of patients were dead or loss to follow-up at 13 months median duration of follow-up [13]. However, pre-ART and ART attrition/retention rate in children have not yet been systematically analysed.

Therefore, in this study we assessed the attrition rates and factors associated with attrition among children (aged ≥ 18 months to < 15 years) enrolled for HIV care since the inception of IHC programme from 2005 to June 2016. The specific objectives of our study were to: (a) describe the demographic and clinical characteristics of children enrolled; (b) retention rates during pre-ART care and ART care at 12, 24, 36, 48 and 60 months after enrolment; and (c) to assess the association between demographic and clinical factors and attrition during pre-ART and ART care periods.

## Methods

### Study design and study population

This was a retrospective cohort study on children (aged 18 months to < 15 years) living with HIV enrolled under the IHC programme from January 2005 to June 2016. Children aged < 18 months were excluded because we could not ascertain their HIV status in our database. This is because, children born to mothers with HIV are also enrolled in this program irrespective of whether they are HIV seropositive or not. Their HIV seropositive or negative status is declared at the age of 18 months and therefore including children aged < 18 months would have resulted to including children who were HIV seronegative in our study which we wanted to avoid.

### Study setting

Myanmar is a low middle-income country (World Bank, 2015) [14] located in the south-east Asia region with the population of 51 million (2014 National Census) [15] with 25% of people living below < USD 2 per day. Also, Myanmar is one of the highest HIV burden country in South East Asia after India, Indonesia and Thailand [16]. HIV was first detected in 1988 and the HIV epidemic is concentrated in people who inject drugs, men who have sex with men, female sex workers and their clients/partners. However, due to the spread of infection from these high risk groups to the general population, approximately 9,300 children have been estimated to have been infected and living with HIV with ~ 7,300 children receiving ART in 2016 [10].

### IHC programme

The NAP together with The Union has been implementing the IHC programme within the public-sector health facilities since 2005 in collaboration with the Ministry of Health and Sports, National Tuberculosis Programme (NTP) and Hospitals form public sectors. Initially, the services began in a single ART clinic in Mandalay region and later expanded to other parts of the country. By the end of 2017 there were 49 IHC clinics (16 ART centers and 33 ART decentralised clinics) covering 37 townships predominantly in upper part of Myanmar with a population coverage of ~ 7 million. Among them, 11 IHC centers (550 bedded Mandalay Children Hospital, 300 bedded Mandalay Teaching Hospital, Specialist Hospital Tharketa, Pakokku General Hospital, Meikhtila Hospital, Myingyan Hospital, Saggaing Hospital, Monywa Hospital, Lashio General Hospital, Taunggyi Women and Children Hospital and Kalaw Hospital) were providing HIV care for children. Children reach IHC program from paediatric hospitals, antenatal/maternity clinics, women and children hospitals, TB clinics, pre-ART clinic run/maintained by NAP, laboratories, community referrals and patients referred from other ART program.

At enrolment, children were assessed for ART eligibility and ART were provided to children as per the prevailing national guidelines at the various time periods 2005–2010, 2010–2014, 2014–2016 as shown in Table 1 [17–19]. In addition, children were also assessed for TB and other opportunistic infections, baseline CD4, nutritional status, haematological parameters and WHO clinical staging.

**Table 1.** Criteria for initiating ART in children (aged < 15 years) with HIV infection under the IHC program in Myanmar.

Time frame	Guidelines for initiating ART
2005–2010	WHO Paediatric Stage 3 or Stage 4 (irrespective of the CD4 Cell count) Based on CD4 cell count if WHO paediatric Stage 1 or 2 and also total Lymphocyte counts guided in stage 2 if CD4 not available
2011–2014	All children < 24 months or if WHO clinical stage is 3 or 4. In clinical stages 1 or 2 treat only if CD4 Cell count is below the age threshold
2015–2016	All children aged < 5 years of age. In children > 5 years, CD4 cell count ≤ 500 cells/cubic ml or WHO stage 3 or 4 (priority to children with advance/severe immunosuppression and CD4 cell count ≤ 350 cells/cubic ml

If the children were not eligible for ART, then they continued in pre-ART care with periodic follow-up once and then progressively increased to once every 3/6 months as determined by the clinician/paediatrician at the IHC clinics. During each follow-up visit, children were assessed for opportunistic infections and ART eligibility. If the children were eligible for ART either at enrolment or during the subsequent follow-up visits, they were initiated on ART therapy (using one of the standard recommended regimens based on clinical status of the child) [17–19]. After ART initiation, they are initially followed up once every 2 weeks in the initial period and then progressively increased to once every 3/6 months if the patient is stable on ART. During each follow-up visit, children were assessed for their clinical status, drug adherence, opportunistic infection, nutritional status, CD4 count and other haematological parameters.

Adherence support systems offered to children by IHC: Children or their care givers were counselled about the importance of adherence by the paediatrician, medical social worker and peer counsellors. If children did not have a family care giver, they were enrolled as an orphan in an orphanage. During follow-up period, if children did not visit the IHC centre on the scheduled visit date, peer network counsellors traced the patients by phone calls and/or home visits and motivated them and their care givers to continue ART care. IHC also provided psychosocial support counselling, disclosure counselling and life skills trainings and also organised yearly fun fairs for children enrolled into the IHC program.

### Recording and reporting systems in the IHC programme

All enrolled children were assigned a unique IHC code and a hard copy of the medical record was maintained in a confidential and secure environment. All data in the hard copy (including follow-up visits) was entered into an electronic database (Excel and IUATLD epi-concept database) at each of the IHC ART centres.

Four primary program outcomes are recorded for each child in their medical records and electronic database: (a) regular follow-up – if the child is alive and under care at the last follow-up date or censoring date; (b) lost to follow up – if the child has missed scheduled visit for ≥ 90 days; (c) died; (d) transferred out – if the child was moved out permanently from IHC program to other service providers.

#### Study outcomes

All eligible children enrolled between January 2005 and June 2016 were assessed for one of following two study outcomes at the end of study period (date of censoring = 30 June 2017).

- Retention – children who are alive and are on follow-up (or on ART) in IHC program at the end of study period, children who were transferred out.
- Attrition – children who were lost to follow-up or have died.

#### Study period, data collection and variables

The study was conducted between March and December 2017. We used the data collected in the IHC program electronic databases to address the various objectives of the study. The variables collected included IHC code, date of enrolment, ART start date, program outcomes, outcome date, gender, and enrolment values for age, weight, height, haemoglobin, CD4 cell count, WHO clinical stage, history of receiving ART before coming to IHC site, hepatitis B virus infection, and the ART regimen.

#### Data analysis and statistics

All statistical analysis has been done in STATA (version 12.1 STATA Corp., College Station, TX, USA). Based on the variables collected, we derived the following additional variables: (a) Pre-ART time period – date of enrolment to the date of outcome or date of ART initiation (whichever was earlier); (b) ART time period – date of ART initiation to date of outcomes/censoring (whichever was earlier); (c) nutritional status (underweight, normal and overweight) from weight, height, age using weight for age Z scores if age< 60 months and BMI if age ≥ 60 months [20–22] d) immunological status into no, mild, advanced, severe immunodeficiency based on absolute CD4 Cell count (if age > 60 months) or on CD4 Cell percent (if age< 60 months) [23]; (e) anaemia status into no, mild, moderate and severe based on haemoglobin values using the WHO recommended anaemia classification system in children. Whenever data were missing for the above variables, we created a separate category/value and used it in our analysis.

We have summarised the demographic, clinical characteristics using numbers and proportions. For calculating the attrition rates during pre-ART care and ART care, we have included in the numerator all those children who had an attrition defining event and in the denominator, we have included the sum total of all the individual pre-ART periods and ART periods, respectively. The pre-ART & ART attrition rates are expressed as number per 100 person years on pre-ART or ART care. We have also calculated the retention (converse of attrition) rates separately at 6, 12, 24, 36 and 60 months using life table method.

We have quantified the independent association between documented demographic and clinical factors and attrition by estimating unadjusted and adjusted hazard ratios using Cox proportional hazards model. We have assessed for the violations of the proportionality assumption using by using the Schoenfeld and scaled Schoenfeld residuals (stphtest command in Stata). We have also provided the smoothened graphs of the hazard function derived using Cox proportional hazards models for the pre-ART and ART attrition to describe the hazard function for the time periods.

## Results

**Figure 1.**
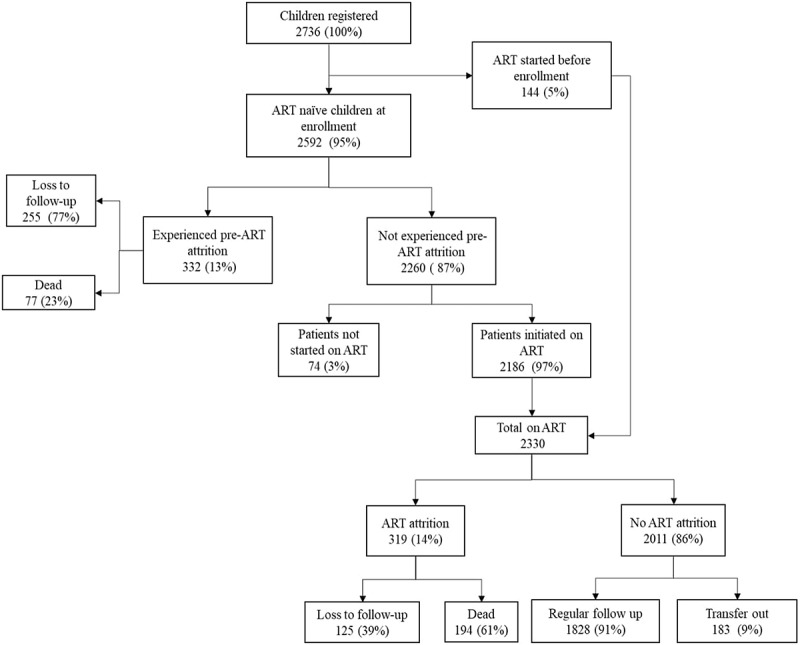
Flow diagram of the study participants and outcomes in children (aged 18 months – < 15 years) enrolled under Integrated HIV Care Program, Myanmar between Jan 2005 – June 2016.

A total of 2,736 children were enrolled into the IHC program and number of patients included in the different analyses and their outcomes are given in the figures Figure 1. The demographic and clinical characteristics of this cohort is given in Table 2.

**Table 2.** Demographic and clinical characteristics of children (aged 18 months – < 15 years) living with HIV enrolled under IHC Program between Jan 2005 – June 2016 (N = 2736).

Patient’s characteristics	Number	(%)
Gender		
male	1450	(53)
female	1286	(47)
Age¶		
18 months to < 5 years	814	(29)
5 years to < 10 years	1197	(44)
10 years to < 15 years	725	(27)
Growth*^¶^		
Normal	423	(15)
Underweight (<-2 Z-score)	2079	(76)
Overweight (> 2 Z-score)	102	(4)
Unknown	132	(5)
Haemoglobin ^¶^		
No anaemia	551	(20)
Mild anaemia	442	(16)
Moderate anaemia	1258	(46)
Severe anaemia	214	(8)
Unknown	271	(10)
WHO clinical stage ^¶^		
Stage 1	697	(25)
Stage 2	596	(22)
Stage 3	1232	(45)
Stage 4	185	(7)
Unknown	26	(1)
Immunological staging (CD4 count/cd4%)^¶^		
No	661	(24)
Mild	311	(12)
Advance	417	(15)
Severe	995	(36)
Unknown	352	(13)
Hepatitis B infection ^¶^		
Positive	66	(3)
Negative	2500	(91)
Unknown	170	(6)
TB history during follow-up		
Present	823	(30)
Absent	1913	(70)

*Weight-for-age Z-score or BMI; ^¶^ at enrolment; HIV-human immunodeficiency virus; IHC – integrated HIV

### Attrition during pre-ART period

Of the 2,736 children enrolled, 144 (5%) children were already on ART at the time of enrolment and were continued on ART. Therefore, they were ineligible for pre-ART attrition. The remaining 2,592 children contributed a combined total of 633,563-person days of pre-ART follow-up time period (median 42 days; range 1–3,514 days). During this period, 333 (13%) had an attrition event [pre-ART attrition rate = 18.9 per 100-person years of follow-up (95% CI: 17.0-21.1)].

**Figure 2.**
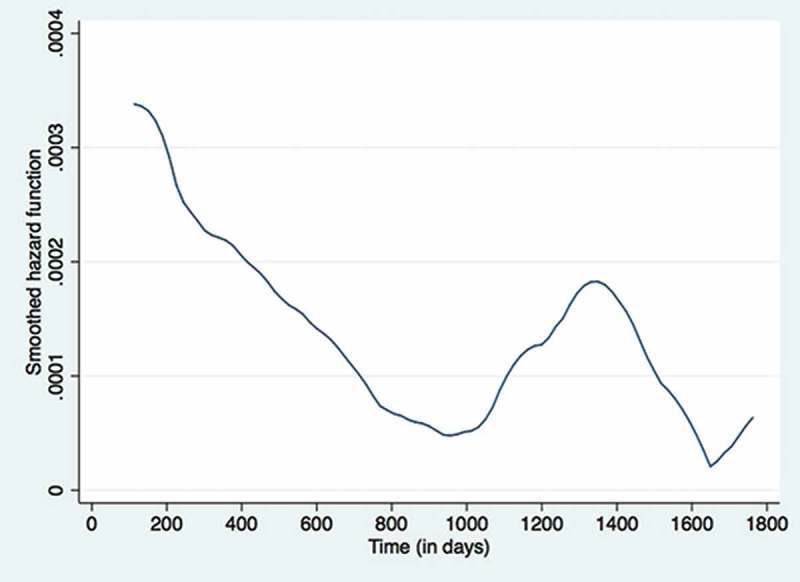
The smoothened ‘hazard function’ for pre-ART attrition in a cohort of HIV positive children (aged 18 months to < 15 years) enrolled under Integrated HIV Care Program, Myanmar between Jan 2005 – June 2016.

The unadjusted and adjusted hazard ratios for demographic and clinical characteristics associated with pre-ART attrition in 2,592 patients are given in Table 3 (no violations of cox proportion assumption). Children who were underweight, or had severe anaemia, or those in WHO stage 3 or 4, or had severe immunological deficiency had higher hazards of pre-ART loss to follow-up when compared to children who were enrolled with relatively normal values for these characteristics. History of TB was associated with lower hazard of pre-ART attrition. The pre-ART ‘hazard function’ derived from the Cox proportional hazards regressions is shown in Figure 2. It indicates that the hazard function for attrition is highest during the initial few days of enrolment and decreases by about 1,000 days (2.7 years) and peaks again at around 1,300 days (3.56 years) to decrease latter on.

**Table 3.** Demographic and clinical factors associated with attrition during pre-ART Care in children (aged 18 months – < 15 years) enrolled under IHC Program between Jan 2005 – June 2016 (n = 2592).

Patient’s characteristics	Number	Attrition (N)	Attrition (per 100 persons-year follow-up) (95% Cl)	Unadjusted hazard ratio (95% Cl)	Adjusted hazard ratio (95% Cl)	P value
**Total**	2592	332	18.9(17.0–21.1)			
**Gender**						
male	1370	177	19.3 (16.6–22.3)	ref	ref	
female	1222	155	18.6 (15.9–21.8)	0.9 (0.8–1.2)	0.9 (0.8–1.2)	0.580
**Age**						
18 months to < 5 years	800	108	16.3 (13.5–19.7)	ref	ref	
5 years to < 10 years	1131	145	18.2 (15.5–21.4)	0.9 (0.8–1.3)	0.9 (0.6–1.2)	0.363
10 years to < 15 years	661	79	27.0 (21.7–33.7)	1.1 (0.8–1.5)	0.9 (0.6–1.1)	0.345
**Growth^a^**						
Normal	408	38	8.3 (6.0–11.4)	**ref**	ref	
Underweight (<-2 Z-score)	1958	205	16.7 (14.5–19.1)	1.5 (1.0–2.1)	1.9 (1.3–2.8)	0.002
Overweight (> 2 Z-score)	100	6	16.3 (7.3–36.3)	1.0 (0.4–2.4)	1.2 (0.5–2.9)	0.650
Unknown	126	83	323.7 (261.1–401.4)	14.0 (9.5–20.1)	5.8 (3.5–9.6)	< 0.001
**Haemoglobin(baseline)**						
No anaemia	515	28	5.6 (3.9–8.2)	ref	ref	
Mild	429	23	6.5 (4.3–9.7)	1.0 (0.6–1.8)	1.0 (0.6–1.8)	0.9
Moderate	1217	77	9.8 (7.9–12.3)	1.4 (0.9–2.2)	1.3 (0.9–2.0)	0.2
Severe	213	21	33.7 (21.9–51.7)	2.9 (1.7–5.3)	2.7 (1.5–4.9)	0.001
Unknown	257	183	335.1 (289.9–387.3)	27.0 (18.0–40.5)	9.2 (5.4–16.7)	< 0.001
**WHO stage (baseline)**						
Stage 1	680	85	12.3 (9.9–15.3)	ref	ref	
Stage 2	596	62	13.6 (10.6–17.4)	0.9 (0.7–1.3)	0.9 (0.7–1.4)	0.8
Stage 3	1153	137	24.2 (20.5–28.6)	1.3 (1.0–1.7)	1.1(0.8–1.5)	0.5
Stage 4	177	29	84.2 (58.5–121.2)	2.4 (1.5–3.6)	1.8 (1.1–2.8)	0.01
Unknown	25	19	302.7 (193.1–474.6)	9.8 (5.9–16.1)	0.4 (0.2–0.7)	< 0.001
**Immunological staging (CD4 count/cd4 %)**						
No	608	61	6.5 (5.1–8.4)	ref	ref	
Mild	297	15	6.3 (3.8–10.5)	0.7 (0.4–1.2)	0.7 (0.4–1.3)	0.205
Advance	407	16	8.9 (5.5–14.5)	0.7 (0.4–1.2)	0.8 (0.4–1.4)	0.415
Severe	976	51	18.9 (14.4–24.9)	1.1 (0.8–1.6)	1.1 (0.8–1.7)	0.483
Unknown	343	189	148.3 (128.6–171.0)	11.2 (8.3–15.1)	2.0 (1.2–3.3)	0.006
**Hepatitis B infection(baseline)**						
Negative	2405	247	14.8 (13.0–16.7)	ref	ref	
Positive	65	6	14.9 (6.7–33.3)	1.1 (0.5–2.5)	1.2 (0.5–2.6)	0.700
Unknown	161	79	198.5 (159.2–247.5)	7.1 (5.5–9.2)	1.9 (1.4–2.6)	
**Previous TB history**						
Absent	1827	276	19.4 (17.3–21.8)	ref	ref	
Present	804	56	17.0 (13.1–22.1)	0.6 (0.4–0.7)	0.7 (0.5–0.9)	0.020

^a^Weight-for-age Z-score or BMI; HIV-human immunodeficiency virus, IHC-integrated HIV care

### Attrition during ART period

Of those who were not classified as pre-ART attrition (n = 2,260 patients), 2,186 (75%) were initiated on ART at IHC. Therefore, the total patients that received ART at IHC were 2,330 (144 patients who were already on ART plus 2,186 patients who were initiated on ART at IHC) (Figure 1). These 2,330 children contributed a combined follow-up period of 2,936,355-person days of ART follow-up (median 1,226 days; range 1–4,138 days). During this period, 319 (14%) had attrition event [ART attrition rate = 3.89 per 100-person years of follow-up (95% CI: 3.48–4.34)].

**Figure 3.**
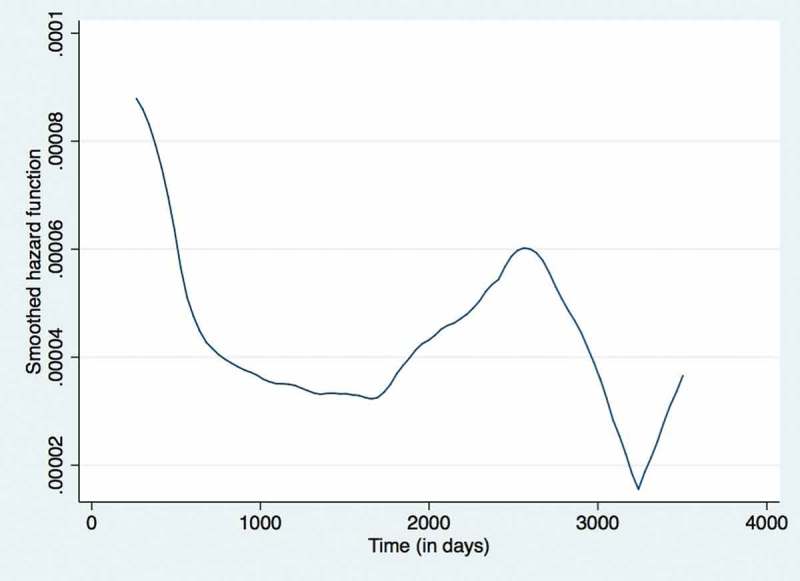
The smoothened ‘hazard function’ for attrition during ART period in a cohort of HIV positive children (aged 18 months to < 15 years) enrolled under Integrated HIV Care Program, Myanmar between Jan 2005 – June 2016.

The unadjusted and adjusted hazard ratios for demographic and clinical characteristics associated with attrition during ART period among 2,330 patients is given in Table 4 (no violations of cox proportion assumption). Females had relatively low hazard rate of attrition when compared to males. Underweight children, those who had moderate to severe anaemia, those on WHO stage 3 or stage 4, and those who had TB had higher hazard rates when compared to children who had normal/absent value for these clinical characteristics. The ‘hazard function’ derived from the Cox proportional hazards regressions for the ART period is shown in Figure 3, The smoothened ‘hazard function’ for attrition during ART period in a cohort of HIV positive children (aged 18 months to < 15 years) enrolled under Integrated HIV Care Program, Myanmar between Jan 2005 and June 2016. It indicates that the hazard function for attrition during ART period was highest during the initial few days of enrolment and decreases by about 1,000 days (2.7 years), remains low till about 1800 days (4.9 years) and increases again at around 2,500 days (6.8 years).

**Table 4.** Demographic and clinical factors associated with attrition during ART Care in children (aged 18 months – < 15 years) enrolled under IHC Program between Jan 2005 – June 2016 (n = 2330).

Patient’s characteristics	Number	Attrition (N)	Attrition rate per 100 persons-year follow-up (95% CI)	Unadjusted hazard ratio (95%CI)	Adjusted hazard ratio (95%CI)	P value
**Total**	2330	316	3.8 (3.4–4.3)			
**Gender**						
Male	1233	178	4.2 (3.6–4.8)	ref	ref	
Female	1097	138	3.4 (2.9–4.1)	0.9 (0.7–1.1)	0.8 (0.6–1.0)	0.050
**Age**						
18 months to < 5 years	683	80	3.2 (2.6–4.0)	ref	ref	
5 years to < 10 years	1016	131	3.5 (2.9–4.1)	1.1 (0.8–1.4)	0.9 (0.7–1.4)	0.919
10 years to < 15 years	631	105	5.2 (4.3–6.3)	1.5 (1.1–2.0)	1.2 (0.8–1.7)	0.347
**Growth^a^**						
Normal	367	28	2.1 (1.5–3.1)	ref	ref	
Underweight	1819	242	3.7 (3.2–4.1)	1.7 (1.2–2.5)	1.3 (0.8–2.0)	0.253
Overweight	96	9	2.6 (1.4–5.1)	1.2 (0.6–2.6)	0.8 (0.4–1.9)	0.670
Unknown	48	37	62.6 (45.3–86.4)	19.4 (11.9–31.8)	11.8 (6.5–21.3)	<0.001
**Haemoglobin (baseline)**						
No anaemia	506	41	2.2 (1.6–3.0)	ref	ref	
Mild	405	35	2.4 (1.7–3.3)	1.1 (0.7–1.7)	1.1 (0.7–1.7)	0.672
Moderate	1146	162	3.8 (3.3–4.5)	1.7 (1.2–2.4)	1.6 (1.1–2.2)	0.014
Severe	193	50	8.3 (6.3–10.9)	3.5 (2.3–5.3)	2.5 (1.6–3.8)	<0.001
Unknown	80	28	20.3 (14.0–29.4)	6.4 (3.9–10.4)	1.5 (0.8–2.9)	0.252
**WHO stage (baseline)**						
Stage 1	570	40	2.3 (1.7–3.2)	ref	ref	
Stage 2	516	39	2.1 (1.6–2.9)	0.9 (0.6–1.6)	0.9 (0.6–1.5)	0.899
Stage 3	1082	185	4.3 (3.7–4.9)	2.2 (1.5–3.1)	1.7 (1.2–2.5)	0.003
Stage 4	156	47	10.4 (7.8–13.8)	4.6 (2.9–6.9)	3.2 (2.0–5.1)	<0.001
Unknown	6	5	139.2 (57.9–334.4)	25.4 (10.0–64.6)	1.4 (0.5–4.1)	0.559
**Immunological staging (CD4 count/cd4%)**						
No	549	29	1,6 (1.1–2.3)	ref	ref	
Mild	288	24	2.4 (1.6–3.5)	1.5 (0.9–2.6)	1.4 (0.8–2.4)	0.236
Advance	398	47	3.3 (2.5–4.4)	2.1 (1.3–3.4)	1.8 (1.1–2.9)	0.015
Severe	939	167	4.6 (3.9–5.4)	3.2 (2.1–4.7)	2.2 (1.5–3.3)	<0.001
Unknown	156	49	11.5 (8.7–15.3)	6.9 (4.3–10.9)	3.9 (2.3–7.0)	<0.001
**Hepatitis B infection(baseline)**						
Negative	2185	271	3.4 (3.0–3.9)	ref	ref	
Positive	59	6	2.6 (1.1–5.7)	0.8 (0.3–1.8)	0.6 (0.3–1.3)	0.176
Unknown	86	39	29.2 (21.3–39.9)	5.7 (4.1–7.9)	5.3 (3.6–7.7)	<0.001
**Previous TB history**						
Absent	1570	174	3.0 (2.6–3.5)	ref	ref	
Present	760	142	5.6 (4.7–6.5)	1.8 (1.4–2.2)	1.3 (1.0–1.6)	0.022

^a^Weight-for-age Z-score or BMI

### Cumulative hazard and retention rates

The cumulative pre-ART and ART hazard rates of this cohort at various time periods is shown in

**Figure 4.**
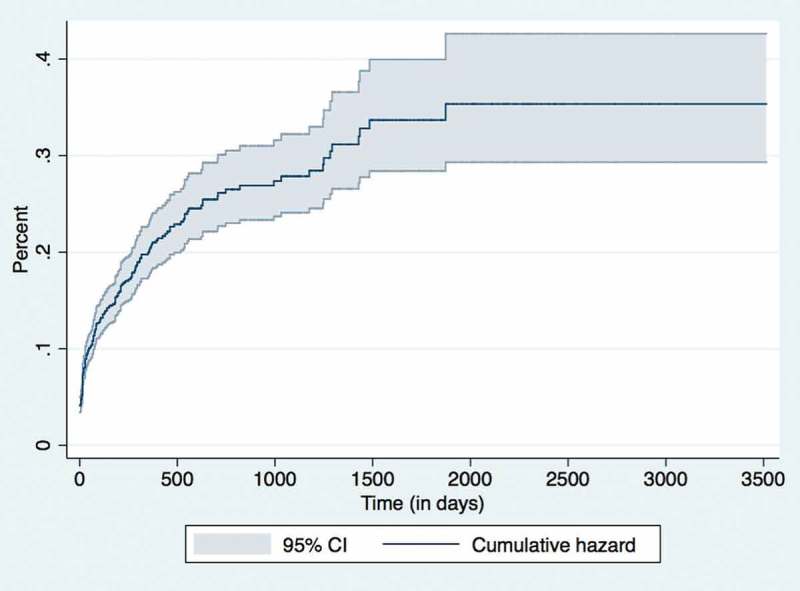
Nelson Aalen Cumulative Hazard graph for Pre-ART attrition in children (aged 18 months – < 15 years) enrolled under IHC Program between Jan 2005 – June 2016 (n = 2,292).

**Figure 5.**
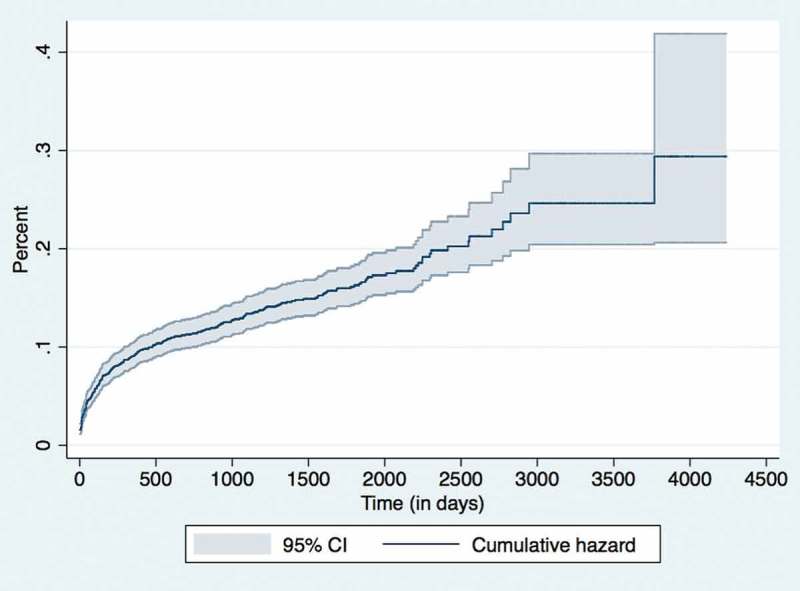
Nelson Aalen Cumulative Hazard graph for ART attrition in children (aged 18 months – < 15 years) enrolled under IHC Program between Jan 2005 – June 2016 (n = 2,330).

Figures 4 and 5, respectively. The corresponding retention rates of this cohort at the end of various time periods (1, 3, 6, 12, 24, 36 and 60 months) is given in Table 5. The 36-month retention rates on pre-ART was 75% (95% CI: 72–78) and ART care 87% (95% CI: 86–88).

**Table 5.** Retention rates of children (aged 18 months – < 15 years) enrolled under IHC Program between Jan 2005 – June 2016.

	Pre-ART period N = 2,592	ART period N = 2,330
Time period (in months)	Cumulative % retained at the end of the time period (95% CI)	Cumulative % retained at the end of the time period (95% CI)
1 months	91 (90–92)	96 (95–97)
3 months	88 (86–89)	94 (93–95)
6 months	86 (84–87)	93 (91–94)
12 months	81 (79–83)	91 (90–92)
24 months	77 (74–79)	89 (88–90)
36 months	75 (72–78)	87 (86–88)
60 months	71 (67–75)	85 (83–86)
120 months	-	78 (75–82)

## Discussion

This is one of the first study from Myanmar describing the experiences of children on ART care over a large time period of about 12 years. This study shows that approximately one in four children on pre-ART care and one in eight children on ART care had either died or lost to follow-up at 36 months. The retention rates during ART (~ 87%) were higher when compared to studies from other countries in Africa and Asia where the 36-month retention rates were 66 and 74%, respectively [9] indicating relatively better organisation and/or delivery of services under the IHC programme.

The major strengths of the study are: (a) a large cohort of nearly 3,000 children (~ 26% of the HIV positive children on ART care in Myanmar) with long time-frame/follow-up period. This has allowed us to measure cumulative attrition rates for various time intervals and also assess hazard function experienced by this cohort both for the short term and long-term; (b) we have used routine programme data, with relatively robust recording and reporting system and have included all eligible children without any exclusion. Therefore, our study findings likely reflect what has happened at the field level.

The major limitations of our study: First, the clinical variables (haemoglobin, CD4 cell count, WHO staging, nutritional status) had missing data which was not ‘at random’, but was strongly associated with attrition (i.e. children with attrition had higher missing data) and also with the clinical factors. Therefore, excluding children with missing data would have introduced selection bias and also reduced the effective sample size. In order to address this, we created a separate category to indicate missing value for each variable, and included children with missing data in our analysis. This prevented us from introducing selection bias. However, we feel that creating a separate category for missing data may have diluted the associations between clinical characteristics and attritions seen in our study (if the associations are indeed truly present in the study population). Second, we used ‘at enrolment’ values of the demographic and clinical characteristics. These values may have changed subsequently for several reasons (for e.g. initiation/non-initiation of ART) prior to the outcome and we have not accounted for this in our analysis. Therefore, our study reflects ‘hazard rates/ratios for attrition’ at ‘enrolment’ for the various demographic and clinical characteristics. Therefore, the programme managers/clinicians must be cautious in applying these hazard rates/ratios if values other than that ‘at enrolment’ are being used. Third, in our setting, other than the demographic and clinical characteristics included in our study, the presence or absence of care giver and their educational status, socio-economic status of the child’s family is known to be associated with higher attrition. Unfortunately, these variables were not captured in our database and therefore we were unable to measure the independent effects of these factors in our study. The magnitude of the association between demographic and clinical factors and attrition seen in our study could be an overestimate or an underestimate depending upon how strongly these unmeasured factors are associated with the measured demographic and clinical characteristics.

Despite these limitations, the study findings have the following implications.

First, the hazard rates for attrition was highest in the initial time periods (especially in the first few days/months) indicating that perhaps children are coming late into the IHC programme or that the initial counselling/care offered is inadequate. To address this, the national AIDS Program and other services providers need to find children living with HIV who did not yet know their status or who cannot access ART services and strengthen the structure of the initial period counselling with the involvement of the peer network. Currently, in Myanmar, an estimated 2000 children are not reaching ART services indicating the need of urgent action to reach the global 90–90-90 targets for diagnosis, ART enrolment and ART retention at the end of 2020 [24]. The reasons for secondary increase in attrition rates at ~ 1500 days (at about 4 years) in the pre-ART period and ~ 2500 days (at about 7 years) during the ART period is unknown. We are unable to speculate any reasons for it at this point in time. This is an area for future research.

Second, of the 651 children who experienced pre-ART or ART attrition, 380 children were ‘lost to follow-up’ suggesting that the loss to follow-up tracing system has to be improved. Anecdotal evidence indicates that children lost to follow-up may have actually died and studies from Africa showed that about or more than half of the patients recorded as lost to follow-up were actually died. Therefore, they may have been benefited if they were traced as soon as they missed the clinic appointment and brought back to the program [25]. Hence, the IHC programme needs to improve the existing lost to follow-up tracing system for children both in pre-ART and ART care preferably on the first day of missing to IHC clinic appointed date.

Third, within different age and sex groups, children with relatively lower baseline haemoglobin levels, immunological levels, nutrition/growth status, higher WHO clinical stages, presence of hepatitis B infection had higher hazards of attrition not only during pre-ART period but also during ART period indicating that clinical condition of the children at enrolment strongly predicts the differences in the attrition rates during both pre-ART and ART periods which is not eliminated by the care given in the IHC programme. Presence of TB was associated with lower hazards of pre-ART attrition and higher hazards of attrition during the ART time period, indicating that children with TB are prioritised for ART initiation (which is in accordance to the existing guidelines) but the subsequent care had not reduced relative differences in the attrition rates between those who had TB and those who did not. These indicate that there is a need to revisit the clinical care strategies provided to children with relatively poorer clinical profiles at enrolment so that these disparities in attrition rates that are seen not only at enrolment, but also during the entire follow-up period can be reduced/minimised.

Last, Myanmar’s National AIDS programme has recently (in 2017) adopted the ‘test and treat’ policy to provide immediate ART therapy to all HIV positive persons (including children) and this should prevent pre-ART attrition and clinical/immunological deterioration of children on ART care and prolong the survival [26]. The effects of introducing this policy can be studied in due course and our study can provide baseline values for future comparisons.

In conclusion, this study showed that the children enrolled into the IHC programme in Myanmar experienced relatively high attrition during pre-ART period and lower attrition during ART periods. The attrition rates were highest during the initial few months of enrolment/initiation of ART and children with poorer clinical/nutritional profiles at enrolment had higher attrition rates and this continued throughout the follow-up period. Improvements in tracing lost-to follow-up children and improving the clinical management of children with poorer clinical profiles, poorer nutritional status at enrolment is likely to reduce the attrition rates.

## Paper context

This study was conducted in Myanmar to assess the attrition (death/loss to follow-up) among children enrolled into an Integrated HIV Care programme from 2005-2016. The attrition rates were highest during the initial few months of enrolment and children with poorer clinical/nutritional profiles at enrolment had higher attrition rates throughout the follow-up period. Improvements in tracing lost-to follow-up children and improving the clinical management of children with poorer clinical/nutritional profiles may reduce the attrition rates.
